# Psychological and cognitive indicators of Takotsubo syndrome

**DOI:** 10.1007/s44192-026-00438-z

**Published:** 2026-04-29

**Authors:** Afaf Hussein, Ali Abd Rabbo

**Affiliations:** Department of Psychology, Faculty of Arts, Benha University, Cairo, Egypt

**Keywords:** Takotsubo syndrome, Psychological indicators, Cognitive functions

## Abstract

**Background:**

Takotsubo syndrome (broken-heart syndrome) is considered a cardiac disorder typically associated with exposure to severe psychological or physical stress, and it clinically resembles acute coronary syndrome. The literature indicates its association with elevated levels of anxiety and depression among patients, in addition to the potential impairment of cognitive functions.

**Aims:**

This study aims to identify the differences in psychological indicators (anxiety & depression) and cognitive functions between patients with Takotsubo syndrome (TTS) and healthy Controls.

**Methods:**

A case–control cross-sectional design was employed. The study sample consisted of twenty patients who were selected from the Health Insurance Hospital in Zagazig, Al-Sharqia Governorate, aged (35.8 ± 8.4) years, and twenty healthy Controls aged (36 ± 9.7) years, who were matched with the patient group in terms of demographic characteristics, including gender, age, educational level, and mental state variables. The study instruments included an initial clinical interview for data collection, the Perceived Stress Scale (PSS-10), Raven’s Progressive Matrices Test, the Hospital Anxiety and Depression Scale (HADS), and the Montreal Cognitive Assessment (MoCA).

**Results:**

The study revealed a significant increase in anxiety and depression levels, along with cognitive impairment in patients with TTS compared to healthy controls, manifested particularly in executive functions, memory, and attention, thereby reflecting a unique interaction between the brain and the heart in this syndrome.

**Conclusions:**

The findings indicate that the psychological and cognitive indicators in patients with TTS are multifactorial in nature, involving an interplay of Cerebral Hypoperfusion, Neurohormonal alterations, and dysfunction of brain networks.

## Introduction

Takotsubo syndrome (TTS) is a cardiovascular disorder that clinically resembles acute myocardial infarction and is typically triggered by intense psychological or physical stress, leading to transient left ventricular dysfunction. The syndrome derives its name from the characteristic shape of the left ventricle, which resembles a Japanese octopus trap (Takotsubo). It is also referred to as broken-heart syndrome, transient apical ballooning syndrome, ampulla-type cardiomyopathy, and stress-induced cardiomyopathy [20, 25, 55].

Although most patients recover within weeks, acute-phase mortality has been reported to reach 4–5%, and long-term complications remain clinically significant. Epidemiological data indicate that TTS accounts for approximately 1–3% of all patients and 5–6% of those undergoing coronary angiography for suspected acute coronary syndrome. Hospitalization rates range from 2.3 to 7.1 per 100,000 individuals annually, with an estimated prevalence of about 2% in the over all population [9, 30, 40].

Despite extensive investigation, the etiology of TTS remains incompletely understood. Accumulating evidence suggests that psychological stressors and excessive catecholamine release play a central role in its pathophysiology. Clinical observations consistently show that acute emotional or physical stress often precedes symptom onset, acting as a precipitating factor for the syndrome [17, 48, 53, 57].

Recent research further indicates that psychological and cognitive processes influence cardiac function through neuroendocrine mechanisms, including increased norepinephrine secretion during the acute phase and altered activity in brain’s stress regulatory regions, such as the amygdala, hippocampus, and prefrontal cortex [26, 33, 56]. However, existing studies have largely emphasized cardiac manifestations, while psychological and cognitive variables—particularly their severity, prevalence, and clinical implications—remain insufficiently explored, limiting definitive conclusions [17, 19].

Accordingly, the present study examines differences in psychological indicators (anxiety and depression) and cognitive functioning between patients with TTS and healthy controls. By adopting a psychocardiological perspective, this research aims to support more comprehensive clinical monitoring and underscore the importance of integrating psychological and cognitive assessment into the evaluation and management of TTS syndrome.The present study tests the following Hypotheses:

### H1

*Statistically significant differences exist in psychological and cognitive indicators between patients with Takotsubo syndrome versus healthy Controls*.

### H2

*Significant correlations exist between psychological and cognitive indicators among patients with Takotsubo syndrome versus healthy Controls*.

## Method

### Study design

A case–control cross-sectional design was employed to compare patients diagnosed with TTS syndrome and healthy controls in terms of psychological and cognitive indicators [20, 31].

### Study sample

#### Psychometric properties sample

This sample consisted of 15 participants, separate from the main sample, who had heart disease and were diagnosed with broken heart syndrome. This sample was used to verify the psychometric properties of the study instruments.

#### Main sample

##### Patient group

Due to the rarity of Takotsubo syndrome and the difficulty in diagnosing it, which requires comprehensive cardiac examinations, the study included all cases meeting the selection criteria, thereby limiting the number of cases. Given the nature of the study and the scarcity of cases, the results were interpreted with methodological caution. This group consisted of 20 patients recruited from the Health Insurance Hospital in Zagazig, Al-Sharqia Governorate. Ethical standards approved for psychological research were strictly observed. The patients received pharmacological treatment, including Aspocid, Dinitra, Ator, Nitromack, and Concor; however, these medications were prescribed solely for clinical identification purposes, and the treating physician confirmed that the administered doses do not have any adverse effects on psychological or cognitive performance.Inclusion and exclusion criteria were defined as follows:

- Inclusion criteria
  Diagnosis by a cardiologist based on the Revised Mayo Clinic Criteria and the International TTS Diagnostic Criteria [8].
- Exclusion criteria
  To ensure the accuracy of the results and to eliminate factors that could influence the outcomes, participants who met the following criteria were excluded:
  - Prior Psychiatric Assessment: Patients who had previously undergone psychological assessments using instruments similar to those used in the current study were excluded to avoid the familiarity effect, which can lead to biased responses.
  - Medical History: Individuals with other cardiac cases or chronic medical cases that could interfere with or influence the results were excluded.
  - Neurological and psychological disorders: Patients diagnosed with neurological or psychological disorders were excluded through careful review of their clinical records and medical history to avoid overlap between these cases’ symptoms and the expected psychological results.
  - Hormonal Factors or Female Hormonal Status: Postmenopausal women were excluded due to the strong association between hormonal changes during this stage and the development of the syndrome.

##### Healthy controls group

This group included 20 healthy Controls who were matched with the patient group in terms of age, educational level, and intelligence. None had a medical history of cardiovascular disease, chronic illnesses, psychiatric or neurological disorders, or organic or functional brain diseases (such as vision or hearing impairments or limb-related problems). They were also free from substance use. Table 1 presents the characteristics of the study sample.

**Table 1:** Comparison of demographic, personal, clinical, and affective characteristics among TTS and HC controls (N = 20)

Variable	Statistics/category	TTS		HC		Test	*p*
Age (years)	Mean ± SD	35.8 ± 8.4		36 ± 9.7		t = 0.087	0.931
	Median (min–max)	36(22–50)		37(20–50)			
Sex	Males	7	35.0%	9	45.0%	X2 = 0.417	0.519
	Females	13	65.0%	11	55.0%		
Occupation	Employed	15	75.0%	14	70.0%	X2 = 0.125	0.723
	Unemployed	5	25.0%	6	30.0%		
Marital status	Single	3	15.0%	4	20.0%	X2 = 3.143	0.208
	Married	12	60.0%	15	75.0%		
	Divorced	5	25.0%	1	5.0%		
Educational level	High school	10	50.0%	10	50.0%	X2 = 0.202	0.896
	Post-secondary non-tertiary education	4	20.0%	3	15.0%		
	Undergraduate	6	30.0%	7	35.0%		
Life stressful events	Constant stress	5	25.0%	–	–	–	–
	Death of a loved one	4	20.0%	–	–		
	Domestic violence	3	15.0%	–	–		
	Quarrels	2	10.0%	–	–		
	Bad news	2	10.0%	–	–		
	Bullying exposure	1	5.0%	–	–		
	Emotional separation	1	5.0%	–	–		
	Financial crisis	1	5.0%	–	–		
	Unidentified	1	5.0%	–	–		
Perceived stress scale	Mean ± SD	–		34 ± 2.6		–	–
	Median (min–max)	–		34.5(29–38)			
Raven’s progressive matrices test	Mean ± SD	102.5 ± 3.4		102.4 ± 3.3		t = 0.143	0.887
	Median (min–max)	103(95–108)		102.5(95–108)			
	Median (min–max)	4.5(2–9)		14.5(10–18)			

SD, standard deviation; T-test was used for comparison between parametric data; Mann–Whitney (U) test was used for comparison between non-parametric data; chi-square (X2) was used for comparison between categorical data; *, *p* < 0.05 is considered significant

### Data collection procedure

All participants were subjected to the following:

#### Interview

All participants underwent an interview for data collection which included background, gender, age, marital status, educational level, and occupation, as well as exposure to stressful events (emotional, physical, or both) lasting one week or more prior to symptom onset. Medical information was obtained by the attending physician and included chronic illnesses, physical symptoms, comorbid health conditions, electrocardiographic (ECG) changes, and confirmation of TTS syndrome diagnosis.

#### Perceived stress scale (PSS-10)

Cohen et al. [11], assesses perceived stress over the past month using 10 items on a 0–4 Likert scale, with higher scores indicating greater stress. It has demonstrated good validity and reliability across diverse populations, including the Arabic version, which has been validated in clinical, medical, and academic contexts [2, 4, 10, 36], supporting its suitability for the current study sample.

#### Raven’s progressive matrices test

Raven [43] adapted for the Egyptian population (ages 5.5–68.4 years), assesses adult intelligence using 36 matrices divided into three difficulty levels: Group A (pattern completion), Group AB (perceiving parts within a whole), and Group B (abstract reasoning based on spatial rules). Each matrix includes six alternatives, scored 0–1 per item. Raw scores were summed, converted to age-based percentile ranks, and then to IQ scores [3]. In this study, reliability was acceptable (Cronbach’s α = 0.752).

#### Hospital anxiety & depression scale (HADS)

Zigmond and Snaith [58] assesses anxiety and depression severity over the past two weeks in clinical populations. It includes 14 items, divided into two subscales: HADS-A (anxiety) and HADS-D (depression), each scored 0–3. Subscale totals range from 0 to 21, with higher scores indicating greater symptom severity: 0–7 = normal, 8–10 = mild, 11–14 = moderate, and 15–21 = severe. In this study, the Arabic version was adapted and standardized. Criterion validity, assessed against the Beck Anxiety and Depression Inventories, yielded a correlation of 0.763 [6, 18], and internal consistency was good (Cronbach’s α = 0.843 for anxiety, 0.852 for depression).

#### Montreal cognitive assessment (MoCA)

According to [37] is a brief Neurocognitive screening tool assessing executive functions, naming, memory, attention, language, abstract thinking, and temporal-spatial orientation. Total scores range from 0 to 30, with ≥ 26 considered normal,lower scores indicate potential cognitive malfunction. In this study, the Arabic version was adapted and standardized. Criterion validity, assessed against the Mini-Mental State Examination (MMSE; [13]), yielded a correlation of 0.782, and internal consistency was satisfactory (Cronbach’s α = 0.764), supporting the tool’s reliability and validity in this population.

### Statistical analysis

**Fig. 1:**
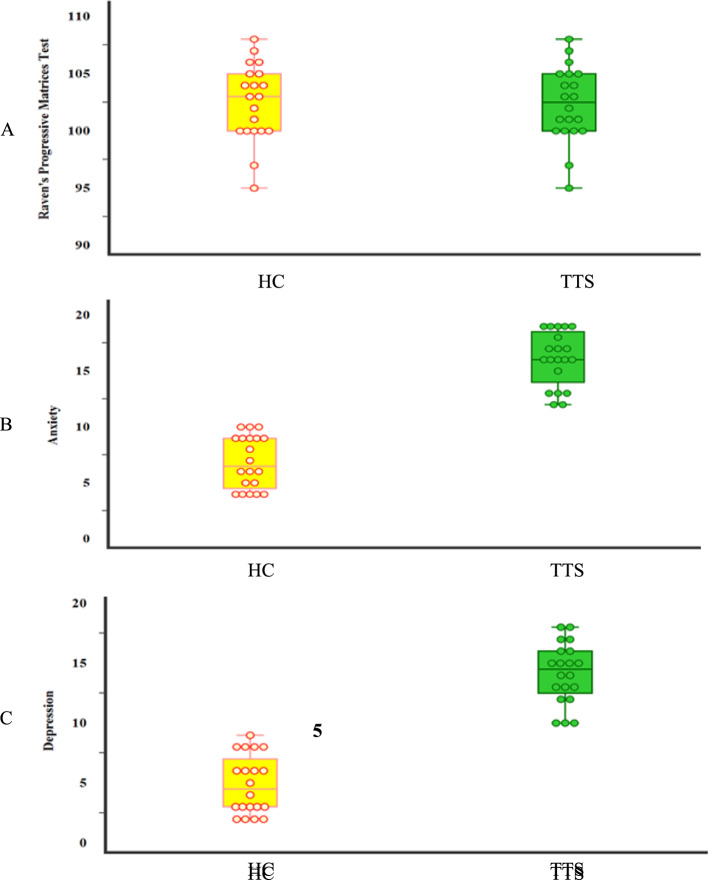
Box plots for comparisons between TTS and HC Controls regarding **A** Raven's Progressive Matrices Test, **B** anxiety scale, and **C** depression scale

**Fig. 2:**
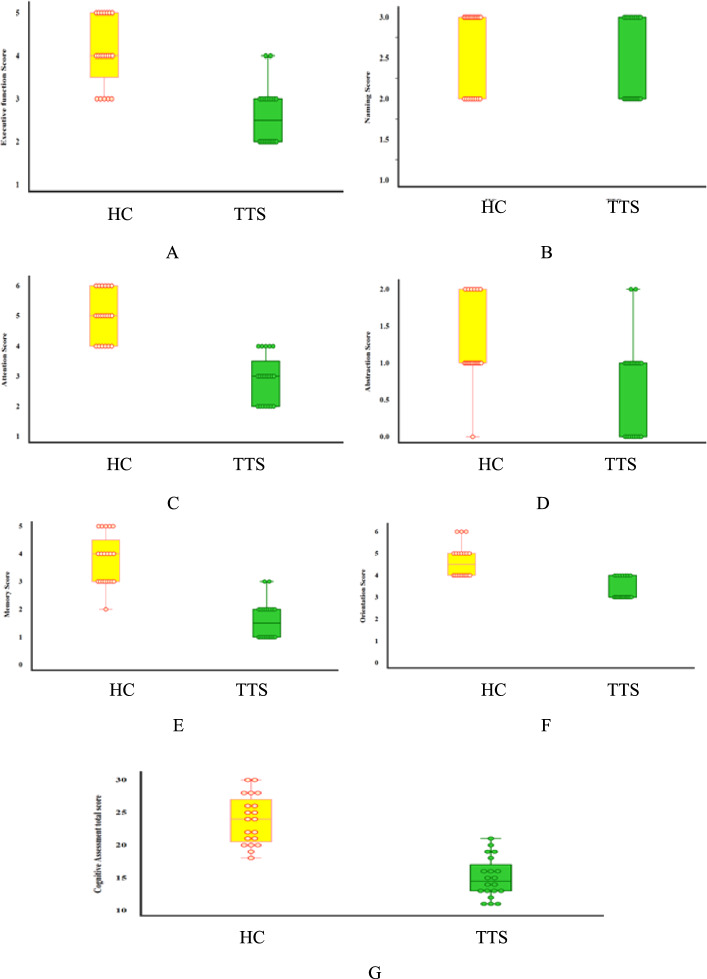
Box plots for comparisons between TTS and HC Controls regrading **A** Executive function Score, **B** naming, **C** attention, **D** abstraction, **E** memory, **F** orientation, and **G** Cognitive Assessment total score

**Fig. 3:**
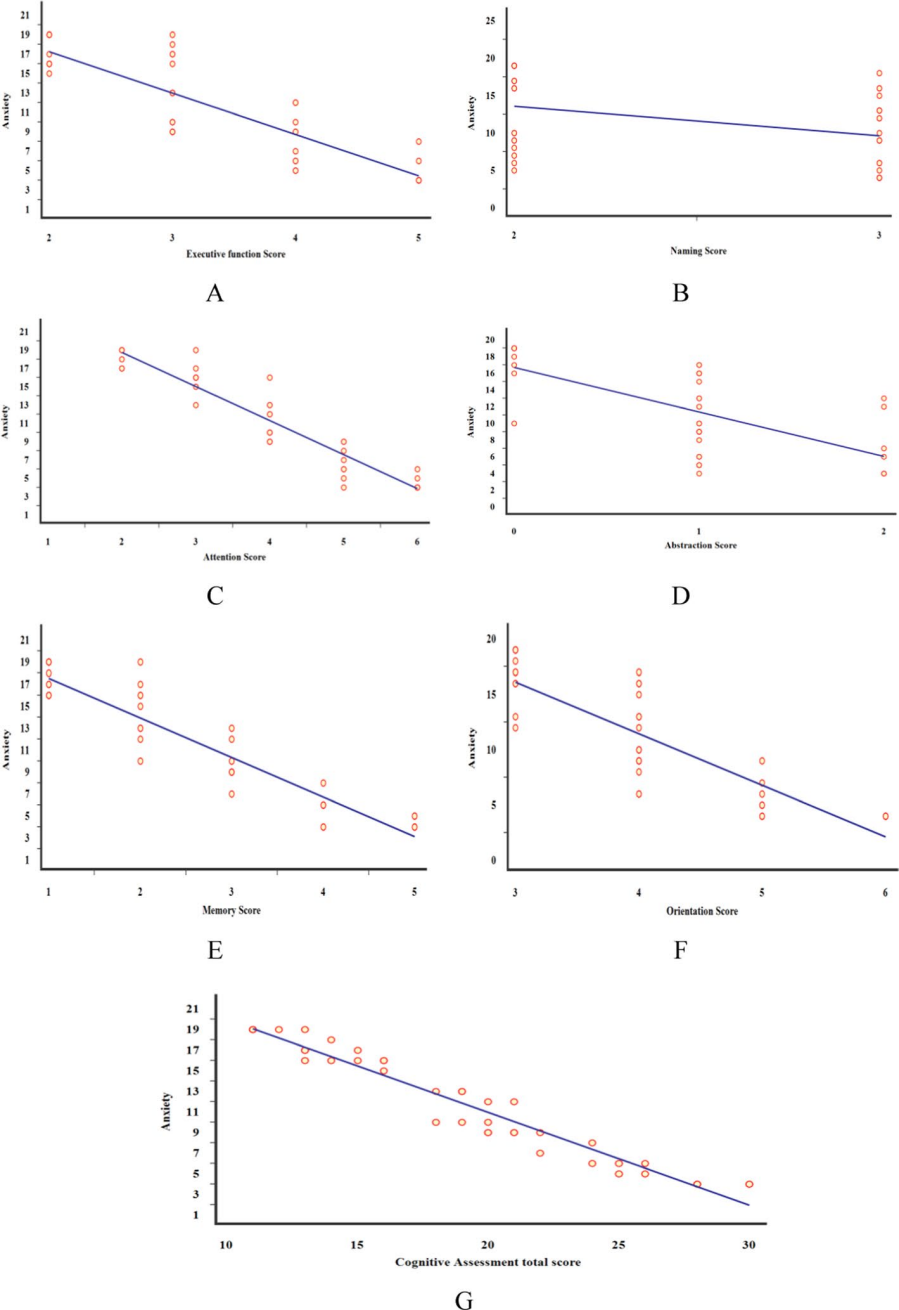
Scatter dot plots for correlations among all studied subjects, between anxiety scale with **A** Executive function Score, **B** naming, **C** attention, **D** abstraction, **E** memory, **F** orientation, and **G** Cognitive Assessment total score

**Fig. 4:**
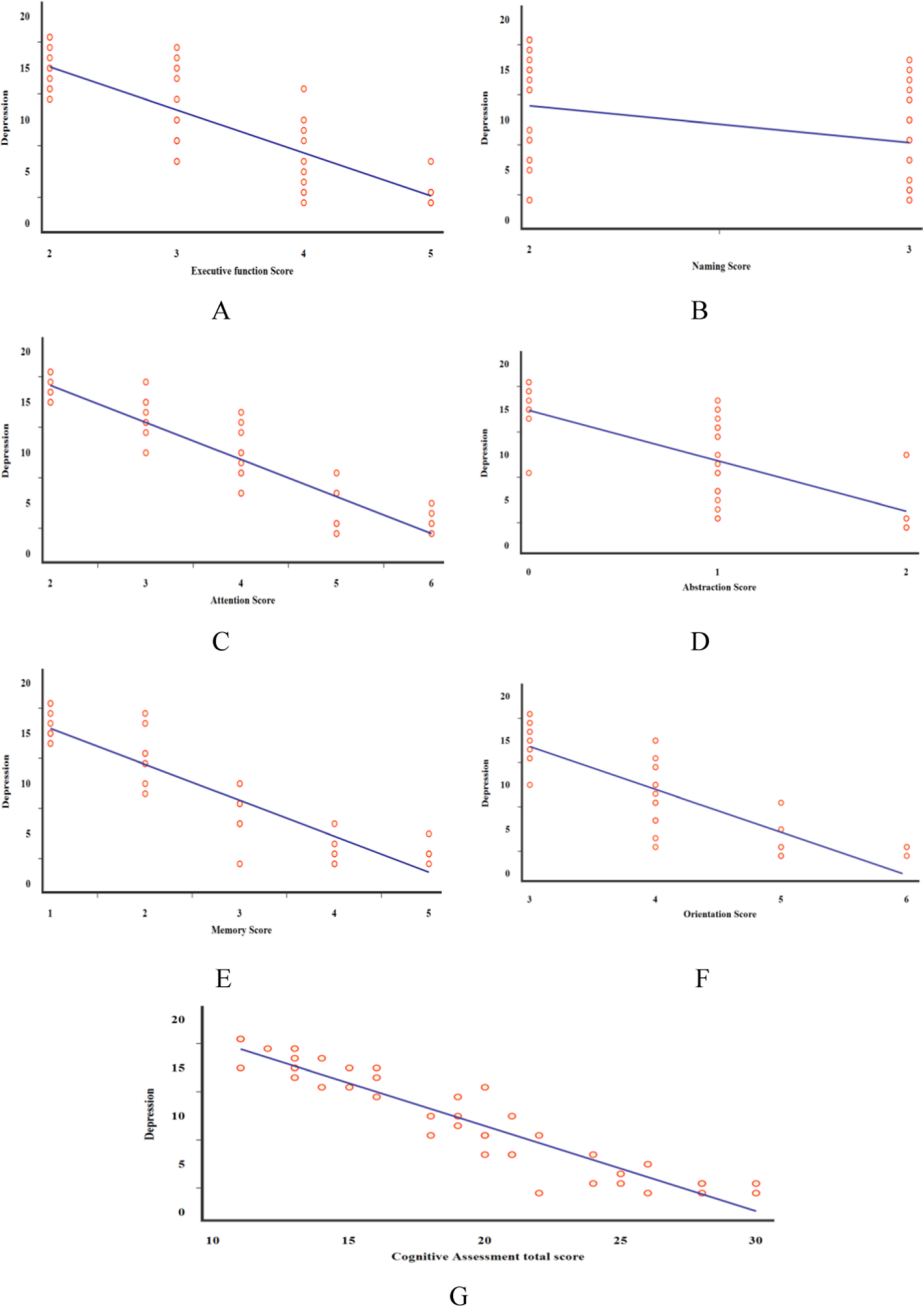
Scatter dot plots for correlations among all studied subjects, between the depression scale with **A** Executive function Score, **B** naming, **C** attention, **D** abstraction, **E** memory, **F** orientation, and **G** Cognitive Assessment total score

**Fig. 5:**
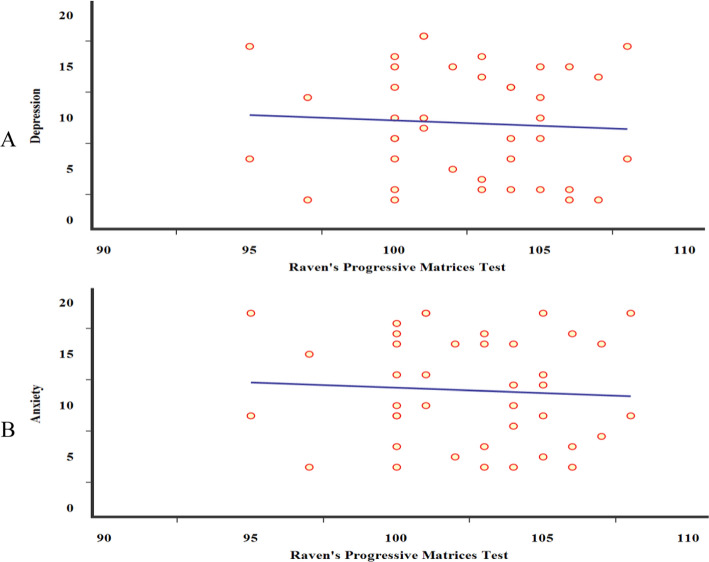
Scatter dot plots for correlations among all studied subjects, between Raven's Progressive Matrices Test with **A** anxiety and **B** depression scales

**Fig. 6:**
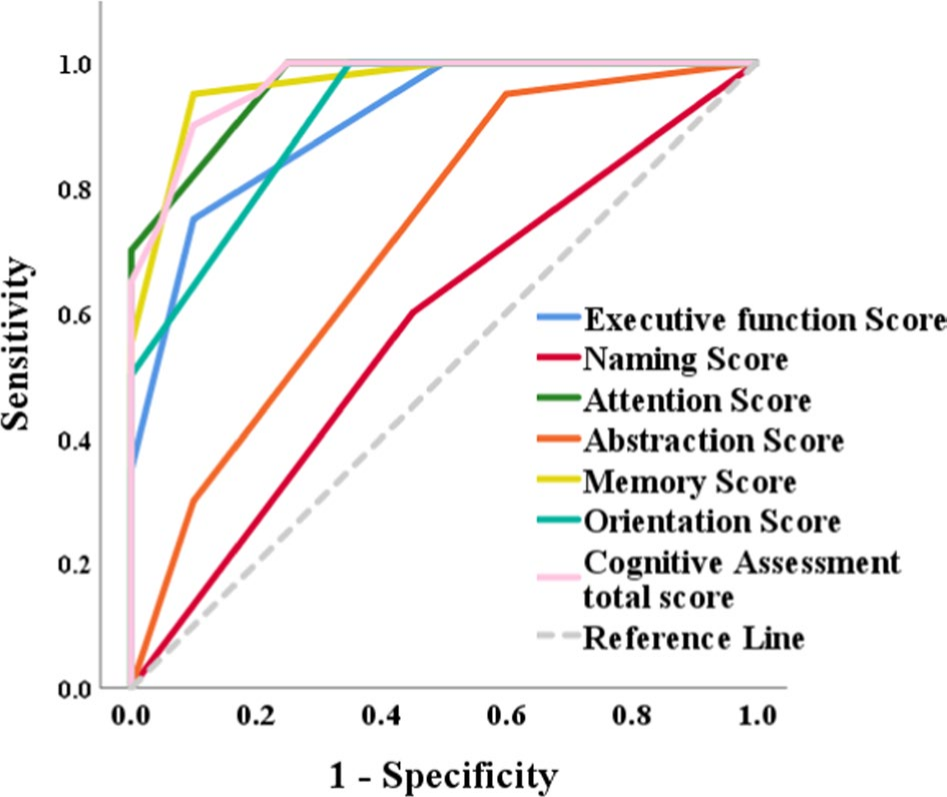
ROC curve of Neuro-Cognitive features for discrimination between TTS and HC Controls

Data were analysed using IBM SPSS Statistics for Windows, Version 25.0 (IBM Corp. [22]). The Shapiro–Wilk test was used to assess normality of numerical variables. Chi-square and Fisher’s exact tests were applied to investigate associations between categorical variables, as appropriate. Independent-samples *T* tests and Mann–Whitney *U* tests were used to compare parametric and non-parametric continuous variables between groups, respectively. It is mentioned in Tables 2 and 3. Pearson’s and Spearman’s correlation coefficients were calculated to examin associations between quantitative variables as it is shown in Tables 4 and 5. Receiver operating characteristic (ROC) curve analysis was performed to evaluate diagnostic sensitivity and specificity, with optimal cut-off points determined by maximizing the area under the curve (AUC) as it is displayed in Table 6. Statistical significance was set at *p* < 0.05 (95% confidence interval) and The Figs. 1, 2, 3, 4, 5, and 6 illustrate the differences between the TTS and HC, the correlations between anxiety, depression, and cognitive functions, as well as the discriminative ability of the psychological and cognitive variables. The ROC curve analysis further demonstrates the strong discriminatory power of most neurocognitive indicators.

**Table 2:** Comparison of affective measurements among TTS and HC controls (N = 20)

Variable	Statistics	TTS	HC	Test	*p*
Hospital anxiety scale	Mean ± SD	16.1 ± 2.4	6.9 ± 2.3	U = 0.0	< 0.001*
	Median (min–max)	16(12–19)	6.5(4–10)		
Depression scale	Mean ± SD	14.1 ± 2.5	4.9 ± 2.4	t = 11.874	< 0.001*
	Median (min–max)	14.5(10–18)	4.5(2–9)		

SD, standard deviation; T-test was used for comparison between parametric data; Mann–Whitney (U) test was used for comparison between non-parametric data; Chi-square (X2) was used for comparison between categorical data; *, *p* < 0.05 is considered significant

**Table 3:** Comparison of neuro-cognitive features among TTS and HC controls (N = 20)

Variable	Statistics	TTS	HC	Test	*p*
Executive function score	Mean ± SD	2.6 ± 0.7	4.1 ± 0.8	U = 38.0	< 0.001*
	Median (min–max)	2.5(2–4)	4(3–5)		
Naming	Mean ± SD	2.5 ± 0.5	2.6 ± 0.5	U = 170.0	0.429
	Median (min–max)	2(2–3)	3(2–3)		
Attention	Mean ± SD	2.9 ± 0.8	5 ± 0.8	U = 15.0	< 0.001*
	Median (min–max)	3(2–4)	5(4–6)		
Abstraction	Mean ± SD	0.7 ± 0.7	1.3 ± 0.6	U = 113.0	0.018*
	Median (min–max)	1(0–2)	1(0–2)		
Memory	Mean ± SD	1.6 ± 0.7	3.8 ± 0.9	U = 14.0	< 0.001*
	Median (min–max)	1.5(1–3)	4(2–5)		
Orientation	Mean ± SD	3.3 ± 0.5	4.6 ± 0.7	U = 35.0	< 0.001*
	Median (min–max)	3(3–4)	4.5(4–6)		
Language	Mean ± SD	1.2 ± 0.4	1.7 ± 0.5	U = 100.0	0.006*
	Median (min–max)	1(1–2)	2(1–2)		
Speech fluency	Mean ± SD	0.2 ± 0.4	0.8 ± 0.4	U = 80.0	0.001*
	Median (min–max)	0(0–1)	1(0–1)		
Cognitive assessment total score	Mean ± SD	15 ± 3.1	23.9 ± 3.7	t = 8.182	< 0.001*
	Median (min–max)	14.5(11–21)	24(18–30)		

SD, standard deviation; T- test was used for comparison between parametric data; Mann–Whitney (U) test was used for comparison between non-parametric data; *, *p* < 0.05 is considered significant

**Table 4:** Correlations between anxiety and depression with other parameters among all subjects

Variable	Anxiety		Depression	
	Correlation coefficient	*p*	Correlation coefficient	*p*
Age	− 0.494	0.001*	− 0.465	0.003*
Perceived stress	− 0.270	0.250	− 0.329	0.157
Executive function	− 0.854	< 0.001*	− 0.817	< 0.001*
Naming	− 0.405	0.009*	− 0.344	0.030*
Attention	− 0.950	< 0.001*	− 0.917	< 0.001*
Abstraction	− 0.590	< 0.001*	− 0.513	0.001*
Memory	− 0.664	< 0.001*	− 0.608	< 0.001*
Orientation	− 0.696	< 0.001*	− 0.711	< 0.001*
Language	− 0.934	< 0.001*	− 0.911	< 0.001*
Speech fluency	− 0.866	< 0.001*	− 0.854	< 0.001*
Cognitive assessment total score	− 0.977	< 0.001*	− 0.940	< 0.001*
Anxiety	–	–	0.970	< 0.001*
Depression	0.970	< 0.001*	–	–

**Table 5:** Association between Socio-Demographic data with Clinical and Affective Characteristics and Neuro-cognitive features among all studied subjects

Variable	Gender		Test	*p*	Occupation		Test	*p*	Marital status			Test	*p*	Educational level			Test	*p*
	Males	Females			Employed	Unemployed			Single	Married	Divorced			High school	Post-secondary non-tertiary education	Undergraduate		
Perceived stress	33.2 ± 2.4	35.3 ± 2.6	t = 1.759	0.096	33.3 ± 2.6	36 ± 1.4	t = 2.213	0.040*	33 ± 2.6	34.5 ± 2.6	33.2 ± 2.9	t = 0.639	0.540	34.8 ± 2.7	34.5 ± 1.7	32.2 ± 2.4	t = 2.261	0.135
	34 (29–37)	36 (30–38)			34 (29–37)	36 (34–38)			34 (30–35)	34.5 (29–38)	35 (30–36)			35.5 (29–38)	35 (32–36)	32 (30–35)		
Raven's Progressive Matrices Test	102.6 ± 3.8	101.9 ± 2.1	t = 0.863	0.394	102.7 ± 3.6	101.4 ± 1.9	t = 1.499	0.142	100.3 ± 5	103.2 ± 2.8	101.6 ± 3.3	t = 1.500	0.236	102.6 ± 2.6	105.5 ± 2.1	99.8 ± 3.3	t = 5.422	0.009*
	103 (95–108)	101 (100–105)			103 (95–108)	100 (100–104)			101 (95–105)	103.5 (100–108)	101 (97–106)			102.5 (100–107)	105.5 (103–108)	101 (95–104)		
Anxiety	16.2 ± 2.5	15.9 ± 2.5	U = 178.5	0.713	16.1 ± 2.6	16 ± 1.9	U = 146.0	0.698	19 ± 0	15.4 ± 2.4	16 ± 2.2	U = 5.507	0.064	15.4 ± 2	17.8 ± 1.5	16.2 ± 3.3	U = 2.498	0.287
	16 (12–19)	16 (12–19)			16 (12–19)	16 (13–18)			19 (19–19)	16 (12–19)	16 (13–19)			16 (12–18)	18 (16–19)	17 (12–19)		
Depression	14.4 ± 2.3	13.7 ± 3	t = 0.301	0.765	14.3 ± 2.6	13.6 ± 2.3	t = 0.231	0.819	16.7 ± 1.5	13.7 ± 2.3	13.8 ± 3	t = 2.637	0.085	13.4 ± 2.2	15.3 ± 1.3	14.7 ± 3.4	t = 0.914	0.410
	15 (10–18)	14 (10–18)			15 (10–18)	14 (10–16)			17 (15–18)	13.5 (10–17)	14 (10–18)			13.5 (10–16)	15 (14–17)	15 (10–18)		
Executive function	2.5 ± 0.7	2.9 ± 0.7	U = 162.0	0.420	2.5 ± 0.7	2.8 ± 0.4	U = 155.0	0.905	2 ± 0	2.8 ± 0.8	2.6 ± 0.5	U = 5.863	0.053	2.6 ± 0.7	2.8 ± 0.5	2.5 ± 0.8	U = 1.147	0.563
	2 (2–4)	3 (2–4)			2 (2–4)	3 (2–3)			2 (2–2)	3 (2–4)	3 (2–3)			2.5 (2–4)	3 (2–3)	2 (2–4)		
Naming	2.5 ± 0.5	2.4 ± 0.5	U = 180.0	0.754	2.5 ± 0.5	2.4 ± 0.5	U = 155.0	0.905	2 ± 0	2.6 ± 0.5	2.4 ± 0.5	U = 5.167	0.076	2.6 ± 0.5	2 ± 0	2.5 ± 0.5	U = 2.031	0.362
	2 (2–3)	2 (2–3)			2 (2–3)	2 (2–3)			2 (2–2)	3 (2–3)	2 (2–3)			3 (2–3)	2 (2–2)	2.5 (2–3)		
Attention	3 ± 0.8	2.7 ± 0.8	U = 185.5	0.859	3 ± 0.8	2.6 ± 0.5	U = 138.0	0.530	2.3 ± 0.6	3 ± 0.9	3 ± 0.7	U = 4.837	0.089	3 ± 0.8	2.5 ± 0.6	3 ± 0.9	U = 1.313	0.519
	3 (2–4)	3 (2–4)			3 (2–4)	3 (2–3)			2 (2–3)	3 (2–4)	3 (2–4)			3 (2–4)	2.5 (2–3)	3 (2–4)		
Abstraction	0.7 ± 0.5	0.7 ± 1	U = 166.5	0.486	0.7 ± 0.6	0.6 ± 0.9	U = 116.5	0.196	0 ± 0	0.9 ± 0.7	0.6 ± 0.5	U = 8.878	0.072	1 ± 0.7	0.3 ± 0.5	0.5 ± 0.5	U = 4.128	0.127
	1 (0–1)	0 (0–2)			1 (0–2)	0 (0–2)			0 (0–0)	1 (0–2)	1 (0–1)			1 (0–2)	0 (0–1)	0.5 (0–1)		
Memory	1.6 ± 0.7	1.6 ± 0.8	U = 166.5	0.486	1.7 ± 0.7	1.4 ± 0.5	U = 143.5	0.633	1.3 ± 0.6	1.7 ± 0.7	1.6 ± 0.9	U = 4.689	0.096	1.7 ± 0.7	1 ± 0	1.8 ± 0.8	U = 3.067	0.216
	2 (1–3)	1 (1–3)			2 (1–3)	1 (1–2)			1 (1–2)	2 (1–3)	1 (1–3)			2 (1–3)	1 (1–1)	2 (1–3)		
Orientation	3.3 ± 0.5	3.4 ± 0.5	U = 187.0	0.902	3.3 ± 0.5	3.4 ± 0.5	U = 131.0	0.402	3 ± 0	3.5 ± 0.5	3.2 ± 0.4	U = 5.845	0.054	3.6 ± 0.5	3 ± 0	3.2 ± 0.4	U = 3.817	0.148
	3 (3–4)	3 (3–4)			3 (3–4)	3 (3–4)			3 (3–3)	3.5 (3–4)	3 (3–4)			4 (3–4)	3 (3–3)	3 (3–4)		
Language	1.2 ± 0.4	1.1 ± 0.4	U = 176.0	0.672	1.2 ± 0.4	1.2 ± 0.4	U = 138.5	0.530	1 ± 0	1.3 ± 0.5	1 ± 0	U = 7.693	0.121	1.3 ± 0.5	1 ± 0	1.2 ± 0.4	U = 0.948	0.622
	1 (1–2)	1 (1–2)			1 (1–2)	1 (1–2)			1 (1–1)	1 (1–2)	1 (1–1)			1 (1–2)	1 (1–1)	1 (1–2)		
Speech fluency	0.2 ± 0.4	0.1 ± 0.4	U = 172.0	0.594	0.2 ± 0.4	0.2 ± 0.4	U = 129.5	0.369	0 ± 0	0.2 ± 0.5	0.2 ± 0.4	U = 2.806	0.246	0.2 ± 0.4	0.3 ± 0.5	0.2 ± 0.4	U = 0.409	0.815
	0 (0–1)	0 (0–1)			0 (0–1)	0 (0–1)			0 (0–0)	0 (0–1)	0 (0–1)			0 (0–1)	0 (0–1)	0 (0–1)		
Cognitive Assessment total score	15 ± 2.9	15 ± 3.6	t = 0.298	0.769	15.1 ± 3.3	14.6 ± 2.5	t = 0.669	0.508	11.7 ± 1.2	16 ± 3	14.6 ± 2.7	t = 4.453	0.019*	16 ± 2.8	12.8 ± 1.7	14.8 ± 3.8	t = 1.411	0.257
	15 (11–20)	14 (11–21)			15 (11–21)	14 (13–19)			11 (11–13)	15.5 (12–21)	15 (11–18)			15.5 (13–21)	12.5 (11–15)	14.5 (11–20)		

SD, standard deviation; T-test was used for comparison between parametric data; Mann–Whitney (U) test was used for comparison between non- parametric data; *, *p* < 0.05 is considered significant

**Table 6:** Validity of different parameters for discrimination between TTS and HC controls

Variable	AUC	95% CI	*p*	Cut off	Sensitivity (%)	Specificity (%)
Raven’s progressive matrices test	0.510	0.328–0.692	0.914	≤ 102.2	52.3	52.7
Anxiety	1	1–1	< 0.001*	> 10	100	100
Depression	1	1–1	< 0.001*	> 9	100	100
Executive function	0.905	0.816–0.994	< 0.001*	≤ 3	90	75
Naming	0.575	0.396–0.754	0.417	≤ 2	55	60
Attention	0.963	0.914–1	< 0.001*	≤ 3	75	100
Abstraction	0.718	0.558–0.877	0.019*	≤ 1	90	30
Memory	0.965	0.915–1	< 0.001*	≤ 2	90	95
Orientation	0.913	0.829–0.996	< 0.001*	≤ 3	65	100
Language	0.750	0.593–0.907	0.007*	≤ 1	80	70
Speech fluency	0.800	0.655–0.945	0.001*	= 0	80	80
Cognitive assessment total score	0.967	0.923–1	< 0.001*	≤ 19	90	90

AUC, area under ROC curve; CI, confidence interval; *, *p* < 0.05 is considered significant

## Results

Table 1 provides a comparative examination of Demographic, personal, Clinical, and Affective features between the TTS group and the healthy Controls (HC) group. No significant differences were observed between the two groups regarding age, sex, occupation, marital status, or educational level (*p* > 0.05). Mean age was comparable between healthy Controls (36.0 ± 9.7 years) and patients with TTS syndrome (35.8 ± 8.4 years; *p* = 0.931).

Among patients with TTS, reported stressful life events included chronic stress (25%), bereavement (20%), domestic violence (15%), disputes and adverse news (10% each), and other emotional or financial stressors (5% each). The mean Perceived Stress Scale score in the TTS group was 34.0 ± 2.6.

The evaluation of cognitive performance using Raven’s Progressive Matrices revealed no significant differences between the groups (*p* = 0.887).

Affective measurements indicated significant differences: the TTS group demonstrated significantly elevated Hospital Anxiety scores (16.1 ± 2.4 vs. 6.9 ± 2.3, *p* < 0.001) and Depression scores (14.1 ± 2.5 vs. 4.9 ± 2.4, *p* < 0.001) in comparison to Controls.

Table 3 contrasts the neurocognitive performance of the TTS group with that of healthy Controls. The TTS group exhibited much poorer scores across almost all neurocognitive assessments. Executive function was significantly diminished in TTS patients (mean 2.6 ± 0.7 vs. 4.1 ± 0.8, *p* < 0.001). Attention (2.9 ± 0.8 vs. 5 ± 0.8, *p* < 0.001), memory (1.6 ± 0.7 vs. 3.8 ± 0.9, *p* < 0.001), abstraction (0.7 ± 0.7 vs. 1.3 ± 0.6, *p* = 0.018), language (1.2 ± 0.4 vs. 1.7 ± 0.5, *p* = 0.006), speech fluency (0.2 ± 0.4 vs. 0.8 ± 0.4, *p* = 0.001), and orientation (3.3 ± 0.5 vs. 4.6 ± 0.7, *p* < 0.001) were all significantly inferior in TTS participants compared to Controls. The naming capacity did not exhibit significant differences between the groups (p = 0.429). The whole cognitive evaluation score was significantly lower in TTS patients (15 ± 3.1) compared to Controls (23.9 ± 3.7, *p* < 0.001).

Table 4 shows bivariate correlations between anxiety and depression scores and the assessed variables across all participants. Both anxiety and depression were significantly correlated with most cognitive domains, including executive function, naming, attention, abstraction, memory, orientation, language, speech fluency, and total cognitive performance. A very strong positive correlation was observed between anxiety and depression scores (*r* = 0.970, *p* < 0.001).

Regarding gender, the investigation of Neuro-cognitive characteristics and psychological metrics between male and female respondents demonstrated no statistically significant differences in all assessed variables (all *p* > 0.05).

In a comparison between employed and jobless individuals, the sole statistically significant difference identified was in Perceived Stress, with unemployed individuals exhibiting greater mean stress levels (36 ± 1.4) compared to employed individuals (33.3 ± 2.6), achieving significance (t = 2.213, *p* = 0.040^*^). No significant differences were observed between the employed and jobless groups in all cognitive testing scores, including Raven’s Progressive Matrices and specific cognitive domains (all *p* > 0.05).

Comparisons across marital status (single, married, divorced) revealed no statistically significant differences in psychological or most cognitive measures. A significant difference was observed only in total cognitive assessment scores, with married participants scoring higher than single or divorced individuals.

Raven’s Progressive Matrices scores differed significantly by educational level, whereas no significant effects of education were found for other cognitive or psychological measures.

Table 6 presents the discriminative performance of study variables in distinguishing patients with TTS syndrome from healthy Controls using receiver operating characteristic (ROC) curve analysis. Most parameters demonstrated strong to perfect discrimination, reflected by high area under the curve (AUC) values (*p* < 0.05). Attention (AUC = 0.963), memory (AUC = 0.965), and total cognitive assessment scores (AUC = 0.967) showed the highest discriminative accuracy. Anxiety and depression scores demonstrated perfect discrimination (AUC = 1.0). In contrast, Raven’s Progressive Matrices (AUC = 0.510) and naming (AUC = 0.575) showed poor discriminative performance.

## Discussion

The findings of this study demonstrate significant differences in psychological indicators and cognitive functioning between patients with TTS syndrome and healthy Controls. Besides the existence of Significant correlations between these indicators.Specifically, patients with TTS exhibited significantly higher levels of anxiety and depression, with mean scores falling within the moderate to severe range. These findings are consistent with previous research demonstrating a strong association between anxiety, depression, and TTS syndrome, suggesting that these psychological factors may contribute to symptom onset and exacerbation, either as pre-existing vulnerabilities or as emotional triggers [17, 47, 49, 50, 55].

Previous studies further report elevated rates of psychological distress among individuals with TTS, including increased prevalence of anxiety and mood disorders, high levels of trait anxiety, and a substantial history of depressive symptoms [35, 46, 54]. A considerable proportion of patients with TTS have been shown to report prior anxiety or depressive disorders and chronic psychological stress, which may increase vulnerability to both cardiovascular and psychiatric conditions compared with healthy Controls [1, 5, 28, 44].

Remarkably, anxiety and depression may function as key risk factors that predispose individuals to TTS syndrome when exposed to psychological stress. These conditions are associated with sympathetic nervous system hyperactivation, excessive catecholamine release, β₂-adrenergic receptor stimulation, and activation of the hypothalamic–pituitary–adrenal (HPA) axis, leading to elevated cortisol levels. Such neuroendocrine alterations are further linked to dysregulation of mood-related neurotransmitters, increased inflammatory cytokine secretion, tachycardia, vascular dysfunction, multi-organ stress responses, and impaired myocardial function, all of which are implicated in the pathophysiology of TTS [15, 16, 23, 54].

These mechanisms are consistent with previous findings linking anxiety, depression, and Type D personality, which is reported as the most prevalent personality profile among patients with TTS [29]. The results also align with neuroimaging evidence demonstrating stress-related functional and structural alterations in brain areas responsible for regulating emotions, especially the amygdala and prefrontal cortex [12, 41]. Notably, amygdala hyperactivity has been shown to predict cardiovascular dysfunction, further supporting a central brain–heart interaction in TTS [52].

Khan et al. [27] indicate that increased expression of specific microRNAs, particularly miRNA-16 and miRNA-26a, among patients with TTS syndrome. These microRNAs are also elevated in individuals with anxiety and depression, further supporting the involvement of a brain–heart axis that may underlie heightened vulnerability to psychological stress and the subsequent development of functional myocardial injury.

The present findings, which agree with the previously mentioned results, indicate that TTS patients exhibited a marked decline in cognitive performance compared to the healthy controls. This decline stresses the remarkable effect of TTS patients on the brain–heart axis. Greatest decreases in most cognitive functions were observed including speech fluency, followed by abstraction, memory, language, attention, executive functions, and orientation. Significant correlations were also observed between levels of anxiety and depression and most cognitive domains, with a Significant correlation between anxiety and depression themselves. These results reflect a pronounced interplay between affective disturbances and cognitive decline in TTS patients, supporting the effect of psychological states influence cognitive performance alongside accompanying cardiac physiological changes.

In addition, These results are consistent with qualitative and neuroimaging evidence showing lower MMSE and MoCA scores, poorer cognitive task performance, reduced activity in the supplementary motor area (SMA) and medial superior frontal gyrus (SFGmed) associated with executive dysfunction, and diminished suppression of the default mode network (DMN), which has been linked to elevated low-density lipoprotein cholesterol (LDL-C). Reduced regional homogeneity (ReHo), amplitude of low-frequency fluctuations (ALFF), and functional connectivity have also been reported in previous studies [21].

These cognitive impairments can be explained by some interrelated mechanisms, most notably cerebral hypoperfusion secondary to impaired cardiac function, whereby reduced ventricular efficiency limits cerebral blood flow and contributes to compromised cognitive performance [14, 32].

Excessive adrenergic stimulation in TTS syndrome, reflected by a catecholamine surge, affects both cardiac and cerebral function, particularly in individuals with pre-existing vascular vulnerability, and may contribute to cognitive disturbances [38, 47].

Neurobiological mechanisms further involve altered neural responses, heightened autonomic nervous system activation following emotional stimuli, and limbic system dysfunction, which disrupts autonomic regulation [24, 34, 40].

Therefore, the findings align with neuroimaging evidence showing reduced functional connectivity and structural and functional alterations in key brain regions implicated in executive and cognitive functions, including the supplementary motor area, central sulcus, lenticular nucleus, hippocampus, precentral cortex, cingulate cortex, prefrontal cortex, precuneus, medial temporal gyrus, amygdala, angular gyrus, and insula. Such neural changes may underlie impairments in planning, decision-making, goal-directed behavior, and emotional regulation [7, 51].

Neurochemical imbalances affecting core cognitive functions, including attention and memory, have been reported in TTS syndrome. Dysregulation of catecholamines (norepinephrine, epinephrine, dopamine), neuropeptide Y (NPY), serotonin, and acetylcholine has been implicated in these deficits, alongside elevated cortisol and copeptin levels reflecting neurohormonal alterations [24, 39, 42, 45]. Taken together, these findings highlight the importance of psychological and cognitive indicators as a central link between emotional stressors and cardiac manifestations in patients with TTS. In the present study, these patients demonstrated marked psychological and cognitive performance compared to healthy Controls, highlighting the importance of comprehensive psychological assessment of patients as an integral part of comprehensive medical care, early screening, and thorough follow-up to identify and manage their cognitive and emotional difficulties.

## Practical implications

The Practical of the present study can be outlined as follows:

- Designing patient education programs to improve patients understanding of TTS, its symptoms, and strategies for coping with associated challenges.
- Designing psychological intervention programs aimed at reducing the severity of psychological symptoms and improving patients quality of life.
- Developing integrated clinical protocols that combine cardiac care with psychological support.
- Guiding future research toward using longitudinal designs to track changes over the long term.

## Limitations

The limitations of The present study could be rendered to the smallness of the sample size, which was drawn from one Health Insurance Hospital in Zagazig, Al-Sharqia Governorate, which may limit the generalizability of the findings. To generalize the results of the study, a larger number of patients in health insurance hospitals in Egypt should be considered.

## Conclusion and recommendations

The findings indicate that the presence of disturbances in psychological and cognitive indicators in patients with TTS is attributable to a complex and multidimensional pathophysiological process, driven by alterations in cerebral circulation, stress-related neurohormonal mechanisms, and functional disruptions within neural connectivity systems. They further highlight that understanding this interplay is essential for developing therapeutic interventions aimed at enhancing psychological and cognitive health alongside cardiac medical care. Furthermore, the study recommends that potential clinical interventions for patients with TTS include stress management programs, cognitive-behavioral therapy, monitoring of anxiety and depression to reduce psychological stress, enhance coping, and mitigate episode severity. Additionally, as the study relied on self-reported questionnaires to assess psychological indicators, future assessments should integrate these with other methods less prone to measurement error. Future approaches could be strengthened by including neuropsychological tests or clinical observation-based assessments, monitoring psychological and cognitive changes using longitudinal designs, and integrating psychological interventions (e.g., stress management, psycho-education) into clinical monitoring.

## Data Availability

The data that support the findings of this study are available from the corresponding author upon reasonable request.
